# Factors associated with the frailty syndrome in elderly individuals living in the urban area^1^

**DOI:** 10.1590/0104-1169.0213.2493

**Published:** 2014

**Authors:** Maycon Sousa Pegorari, Darlene Mara dos Santos Tavares

**Affiliations:** 2 Doctoral student, Universidade Federal do Triângulo Mineiro, Uberaba, MG, Brazil. Scholarship holder from Fundação de Amparo à Pesquisa de Minas Gerais (FAPEMIG), Brazil; 3 PhD, Associate Professor, Departamento de Enfermagem em Educação e Saúde Comunitária, Instituto de Ciências da Saúde, Universidade Federal do Triângulo Mineiro, Uberaba, MG, Brazil

**Keywords:** Frail Elderly, Urban Population, Odds Ratio, Health of the Elderly, Health Status

## Abstract

**OBJECTIVE:**

to identify the occurrence and factors associated with pre-frailty and frailty conditions among elderly individuals.

**METHODS:**

this cross-sectional, observational and analytical household survey was conducted with 958 elderly individuals living in the urban area. The Brazilian version of the Functional Assessment Questionnaire and Multidimensional Scales (Depression, Katz and Lawton brief geriatric versions) were used, together with the Phenotype of Frailty developed by Fried. Descriptive analysis was performed along with a bivariate and multinomial logistic regression model (p<0.05).

**RESULTS:**

a total of 313 (32.7%) non-frail elderly individuals were found in addition to 522 (55.4%) pre-frail and 128 (12.8%) frail individuals. Factors associated with pre-frailty and frailty, respectively, included: being 70├ 79 years old and 80 years old or older; using 1├ 4 medications and 5 or more; greater number of morbidities, functional disability for instrumental activities of daily life, and negative self-perception. The absence of a partner was associated with pre-frailty while hospitalization in the last year, functional disability for basic activities of daily life and indication of depression were associated with frailty.

**CONCLUSION:**

pre-frailty and frailty conditions presented a percentage higher than that reported by Brazilian studies and are associated with health-related variables. These variables can be prevented with interventions directed to the health of elderly individuals.

## Introduction

Frailty among elderly individuals can be defined as a geriatric clinical syndrome that involves a physiological state of increased vulnerability to stressors that result in decreased physiological reserves and deregulation of multiple systems. It is supported by a triad of changes related to the aging process: sarcopenia, neuroendocrine deregulation, and immune system dysfunction^(^ 1 ^-^ 2 ^)^.

From the operational point of view, it is understood as a phenotype of frailty, proposed by Fried et al.^(^ 1 ^)^, which includes five components: unintentional weight loss, self-reported fatigue and/or exhaustion, decreased muscle strength, slow walking speed, and low level of physical activity. Hence, the presence of one or two criteria characterizes the pre-frailty condition, while three or more criteria characterize frailty^(^ 1 ^)^.

Frailty is considered a predictor of adverse outcomes, such as: comorbidities, falls, the use of healthcare services, health conditions, institutionalization, impairment, negative impact on quality of life, mortality, and its prevalence is particularly relevant for the field of public health^(^ 1 ^-^ 3 ^)^.

Brazilian and international studies have reported different prevalence rates, ranging from 6.9% to 40.6% among frail elderly individuals and 46.3% to 60.1% among pre-frail individuals. Associated factors include: being female, advanced age, low level of education and income; absence of a partner, living alone, negative health perception, functional disability, comorbidities, hospitalization, and indication of depression^(^ 1 ^,^ 4 ^-^ 8 ^)^.

In this sense, the frailty syndrome should be a target of investigations and interventions given its impact on elderly individuals, their families and the society as a whole^(^ 2 ^)^. Despite recent initiatives^(^ 4 ^-^ 5 ^,^ 8 ^)^, there are few Brazilian studies assessing this condition and its associated factors taking into account pre-frail individuals. Differently from other studies conducted in Brazil addressing 65 years old or older individuals, this study's object of study focuses on 60 years old or older individuals. Deeper understanding regarding this syndrome can support the implementation of public policies and planning of strategic healthcare actions directed to this population and contribute to investigations in the Brazilian context to identify frail elderly individuals, considering loco-regional specificities.

This study's objective was to identify the occurrence and factors associated to the pre-frailty and frailty conditions among elderly individuals living in the urban area.

## Methods

Household survey with analytical, observational and cross-sectional design conducted in 2012 with 958 elderly individuals living in the urban area of the city of Uberaba, MG, Brazil.

A representative sample of the elderly population living in urban Uberaba, MG, Brazil with 2,149 individuals was aimed at. The sampling computation considered 95% of confidence, 80% of test power, 4.0% of margin of error for interval estimates and an estimated proportion of π=0.5. Stratified proportional sampling was used, so that the various neighborhoods were considered strata. 

The selection criteria included: being 60 years old or older, living in the urban area, not showing cognitive decline, being able to walk, being allowed to use an assistive device (e.g. cane, crutch, or walker), and providing written consent. Exclusion criteria were: not being located after three attempts, being hospitalized, or having neurological diseases that impeded the assessments. 

A total of 958 elderly individuals met the inclusion criteria. Exclusion or losses included individuals who refused to participate (37); were hospitalized (14); had died (266); were not found after three consecutive visits (376); presented cognitive decline (160); other reasons (252); lived in the same household (64); and did not complete all the tests (22).

Previously trained undergraduate and graduate students collected the data. Interviews were held during a single meeting at the individuals' homes, divided into two stages: face-to-face semi-structured interview and anthropometric assessment plus physical performance tests. Before the interview, a cognitive assessment was conducted, using the translated version of an instrument validated in Brazil that considers education at the cut-off points for identifying cognitive deficit^(^ 9 ^)^.

The dependent variable, frailty syndrome, was identified through five items, described as components of the frailty phenotype proposed by Fried et al.^(^ 1 ^)^, as follows: (1) unintentional weight loss assessed by the question: "Have you unintentionally lost more than 4.5 kg or 5% of your body mass (that is, without diets or exercises) in the last year?"; (2) self-report of exhaustion and/or fatigue, measured through items 7 and 20 of the Brazilian version of the Depression Scale by CES-D. The individuals scoring 2 or 3 in any of the questions met the criterion for frailty in this item^(^ 10 ^)^; (3) decreased muscle strength, verified on the basis of grip strength, measured through a manual hydraulic dynamometer type JAMAR, model SAEHAN^(r)^ SH5001 - 973, following the recommendations of the American Society of Hand Therapists. Three measures were obtained at an interval of one minute and are presented in kilogram/strength (Kgf). The average value is considered, adopting the cut off points proposed by Fried et al.^(^ 1 ^)^; (4) slow walking speed, in which time (in seconds) spent to walk a distance of 4.6 meters was considered. The individual walked a total distance of 8.6 meters while the two initial meters and the two final meters were not considered for the computation of the time spent walking. Three measures were taken and their average value was considered; this measure is presented in seconds. A professional stopwatch brand Vollo^(r)^, model VL-1809 was used as the standard, together with the cut-off points proposed by Fried et al.^(^ 1 ^)^; and (5) low level of exercise verified by the long version of the International Physical Activity Questionnaire (IPAQ), adapted for elderly individuals^(^ 11 ^)^. The classification used for this component considered active those individuals who exercised 150 minutes or more weekly. Inactive individuals where considered those who spent 0 to 149 minutes per week with physical activities. Individuals who met three or more of these criteria were classified as frail, those who met one or two items were considered pre-frail, and those whose scores were negative in the tests were considered robust or non-frail^(^ 1 ^)^.

The following were selected for the exploratory variables: (1) socio-economic and demographic characteristics: sex, race, age, marital status, housing, schooling, individual income measured through the Brazilian version of the Multidimensional Functional Assessment Questionnaire (BOMFAQ)^(^ 12 ^)^; (2) clinical health indicators: morbidities, number of regular medications, health perception verified through a question contained in BOMFAQ^(^ 12 ^)^ with alternative answers on a Likert scale: "In general, would you say your health is (very poor, poor, fair, good, excellent)?", self-reported smoking (yes, no) and hospitalization in the last year (yes, no); (3) indication of depression measured through the Geriatric Depression Scale (GDS-15), considering the cut-off point above 5^(^ 13 ^)^; and (4) functional disability was verified through self-report with the application of Katz's^(^ 14 ^)^ scale for basic activities of daily living (BADL) and Lawton's scale for instrumental activities of daily living (IADL)^(^ 15 ^)^.

A spreadsheet was created to store data in Microsoft Office 2007 Excel^(r)^. Two people entered the collected data with dual input and the existence of inconsistencies was verified in two bases. After the necessary corrections, together with the original interview, the database was transferred to the Statistical Package for Social Sciences (SPSS) version 17.0 for analysis.

The categorical variables were analyzed through absolute frequencies and percentages and average and standard deviation were used for the numerical variables. In order to verify factors associated with pre-frailty and frailty, preliminary bivariate analysis was applied, using tests for measures of association (Coefficient Phi, Cramer's V and Kendall's tau-b) in contingence tables to verify tendencies among the exploratory variables (socio-demographic and economic, clinical, indication of depression, functional capacity and self-reported morbidities) with the dependent variable (level of frailty). The tests were considered significant when *p*<0.10.

According to the inclusion criteria (*p*<0.10), the outcome variables were included in the multivariable regression model. In this stage, the number of self-reported morbidities variable was quantitative. Factors associated with pre-frailty and frailty were identified through multivariable analysis with odds ratio of prevalence through the multinomial logistic regression model (saturated model), considering a level of significance of 5% (*p*<0.05) and confidence interval (CI) of 95%.

The study received approval from the Institutional Review Board at the Federal University of Triângulo Mineiro (Protocol 2265/2012), in accordance with Brazilian Council of Health Resolution 196/96, and the subjects signed free and informed consent forms.

## Results

The average age among the 958 participants was 73.77 years old (sd=±6.78), most were women (64.4%), aged between 70 and 80 years old (50.4%), Caucasian (56.6%), from 1 to 4 years of schooling (55.5%), and individual income of 1 time the minimum wage (51.2%). A total of 12.8% (n=123) of the participants were frail, 54.5% were pre-frail (n=522) and 32.7% (n=313) were not frail.

With regard to associated factors, the variables of the preliminary bivariate analysis submitted to multivariable analysis according to the criterion of inclusion (*p*<0.10) were: being a woman (*p*=0.039), aged 80 years old or older (*p*<0.001), without a partner (*p*=0.012), living alone (*p*=0.084), illiteracy (*p*<0.001), individual income up to 1 time the minimum wage, negative health perception (*p*<0.001), using 5 or more medications (*p*<0.001), hospitalization in the last year (*p*<0.001), indication of depression (*p*<0.001) dependence to perform BADL (*p*<0.001) and IADL (*p*<0.001), and 5 or more morbidities (*p*<0.001), Tables 1 and 2.

**Table 1:** - Distribution of socio-economic and demographic variables among levels of frailty. Uberaba, MG, Brazil, 2012

Variables		Frailty			Pre- frailty			No frailty			Total			p*
		n	%		n	%		n	%		n	%		
Sex														0.039
	Male	32	26.0		187	35.8		122	39.0		341	35.6		
	Female	91	74.0		335	64.2		191	61.0		617	64.4		
Age groups														<0.001
	60├ 70 years old	23	18.7		128	24.5		127	40.6		278	29.0		
	70├ 80 years old	51	41.5		286	54.8		146	46.6		483	50.4		
	80 years old or older	49	39.8		108	20.7		40	12.8		197	20.6		
Race														0.170
	Caucasian	81	65.9		293	56.1		167	53.4		541	56.5		
	Afro descendant	10	8.1		62	11.9		43	13.7		115	12.0		
	Mixed	27	22.0		150	28.7		94	30.0		271	28.3		
	Asian	2	1.6		14	2.7		7	2.2		23	2.4		
	Indian	3	2.4		2	0.4		2	0.6		7	0.7		
Marital status														0.012
	No partner	69	56.1		321	61.5		163	52.1		553	57.7		
	Partner	54	43.9		201	38.5		150	47.9		405	42.3		
Housing														0.084
	Lives alone	14	11.4		103	19.7		62	19.8		179	18.7		
	Have company	109	88.6		419	80.3		251	80.2		779	81.3		
Schooling														<0.001
	Illiterate	37	30.1		118	22.6		57	18.2		212	22.1		
	1┤4 years	70	56.9		293	56.1		169	54.0		532	55.5		
	5 or more	16	13.0		111	21.3		87	27.8		214	22.3		
Individual income^†^														0.009
	No income	10	8.1		37	7.1		26	8.3		73	7.6		
	Up to 1 time the minimum wage	73	59.3		278	53.5		138	44.2		489	51.2		
	1├ 3 times the minimum wage	35	28.5		161	31.0		120	38.5		316	33.1		
	More than 3 times the minimum wage	5	4.1		44	8.5		28	9.0		77	8.1		

* Coefficient Phi and Kendall's tau-b, *p*<0.05

† Minimum wage in 2012 in Brazil (R$ 622.00/month).

**Table 2:** - Distribution of clinical and health variables, functional ability and indication of depression. Uberaba, MG, Brazil, 2012.

Variables		Frailty			Pre-frailty			No frailty			Total			p*
		n	%		n	%		n	%		n	%		
Health perception^†^														<0.001
	Negative	79	64.2		277	53.2		107	34.2		463	48.4		
	Positive	44	35.8		244	46.8		206	65.8		494	51.6		
Morbidities														<0.001
	None	0	0		7	1.3		14	4.5		21	2.2		
	1├ 4	32	26.0		184	35.2		154	49.2		370	38.6		
	5 or more	91	74.0		331	63.4		145	46.3		567	59.2		
Use of medications														<0.001
	None	0	0		2	0.4		0	0		2	0.2		
	1├ 4	56	46.7		280	59.1		181	74.2		517	61.7		
	5 or more	64	53.3		192	40.5		63	25.8		319	38.1		
Smoking														0.786
	Yes	17	13.8		62	11.9		41	13.1		120	12.5		
	No	106	86.2		460	88.1		272	86.9		838	87.5		
Hospitalization in the last year														<0.001
	Yes	40	32.5		92	17.6		32	10.2		164	17.1		
	No	83	67.5		430	82.4		281	89.8		794	82.9		
Basic Activities of daily life														<0.001
	Dependent	14	11.4		19	3.6		2	0.6		35	3.7		
	Independent	109	88.6		503	96.4		311	99.4		923	96.3		
Instrumental Activities of daily life														<0.001
	Dependent	114	92.7		374	71.6		180	57.5		668	69.7		
	Independent	9	7.3		148	28.4		133	42.5		290	30.3		
Indication of depression														<0.001
	Yes	55	44.7		137	26.2		50.0	16.0		242	25.3		
	No	68	55.3		385	73.8		263	84.0		716	74.7		

* Cramer's V, Coefficient Phi and Kendall's tau-b, *p*<0.05

† Negative (very poor/poor/fair) and Positive (good/excellent).

The variables included in the multivariate model of multinomial logistic regression are presented in Table 3. Factors associated with pre-frailty include: being aged between 70 and 79 years old (*p*<0.001) and 80 years old or older (*p*<0.001), not having a partner (*p*<0.001), using from 1 to 4 medications (*p*=0.035) and 5 or more medications (*p*=0.002), having a higher number of self-reported morbidities (*p*=0.017), functional disability to perform IADL (*p*<0.001), and having a negative health perception (*p*=0.002). The following associated factors were found for the condition of frailty: being between 70 and 79 years old (*p*=0.022) and 80 years old or older (*p*<0.001), having been hospitalized in the last year (*p*<0.001), taking from 1 to 4 medications (*p*=0.041) and 5 or more medications (*p*=0.006), number of self-reported morbidities (*p*=0.002), functional disability to perform BADL (*p*=0.009), functional disability for IADL (*p*<0.001), indication of depression (*p*=0.033), and negative health perception (*p*=0.023), Table 3.

**Table 3:** - Final model of the multinomial logistic regression for the variables associated with the frailty and pre-frailty condition. Uberaba, MG, Brazil, 2012

Variables		Pre-frailty				Frailty		
		OR*	95%CI^†^	p^‡^		OR*	95%CI^†^	p^‡^
Age groups								
	60├ 70 years old	1				1		
	70├ 80 years old	2.09	1.48-2.96	<0.001		2.06	1.10-3.58	0.022
	80 years old or older	2.42	1.49-3.90	<0.001		5.98	2.96-12.09	<0.001
Sex								
	Male	1				1		
	Female	0.70	0.49-1.01	0.055		1.18	0.65-2.13	0.579
Marital Status								
	No partner	1.84	1.27-2.67	0.001		1.28	0.72-2.26	0.392
	Partner	1				1		
Housing								
	Lives alone	1				1		
	Has a companion	0.88	0.58-1.36	0.585		0.58	0.27-1.22	0.155
Schooling								
	Illiterate	0.95	0.58-1.56	0.846		1.19	0.53-2.65	0.665
	1┤4 years	0.96	0.65-1.42	0.830		1.03	0.51-2.07	0.925
	5 or more	1				1		
Income^§^								
	Yes	1.52	0.66-3.45	0.323		2.59	0.60-11.21	0.202
	Up to 1 time the minimum wage	1.13	0.61-2.08	0.688		2.06	0.65-6.51	0.217
	1├ 3 times the minimum wage	0.79	0.43-1.45	0.449		1.56	0.49-5.04	0.448
	More than 3 times the minimum wage and more	1				1		
Hospitalization								
	Yes	1.45	0.92-2.30	0.109		2.89	1.60-5.24	<0.001
	No	1				1		
Use of medication								
	None	1				1		
	1├ 4	1.62	1.03-2.52	0.035		3.67	1.05-12.78	0.041
	5 or more	2.36	1.36-4.10	0.002		6.06	1.65-22.17	0.006
Number of comorbidities		1.08	1.01-1.15	0.017		1.15	1.05-1.26	0.002
Disability to perform BADL								
	Yes	4.36	0.96-19.83	0.057		8.68	1.73-43.51	0.009
	No	1				1		
Disability to perform IADL								
	Yes	1.47	1.06-2.05	0.020		5.31	2.46-11.41	<0.001
	No	1				1		
Indication of depression								
	Yes	1.07	0.71-1.61	0.738		1.80	1.04-3.12	0.033
	No	1				1		
Health perception								
	Negative	1.67	1.19-2.33	0.002		1.82	1.08-3.05	0.023
	Positive	1				1		

* Odds Ratio

† Confidence interval

‡ *p*<0.05; 1: Category of reference

§ Minimum wage in 2012 in Brazil (R$ 622.00/month)

## Discussion

Elderly individuals in pre-frailty and frailty condition represent an expressive percentage of the individuals aged 60 years old or older addressed in this study; more than half of the participants were in the condition of pre-frailty. Recent Brazilian studies show lower prevalence, such as those conducted by Rede FIBRA in Campinas, SP (51.8%; 9%)^(^ 4 ^)^ and in Belo Horizonte, MG (46.3%; 8.7%)^(^ 5 ^)^. Other studies report higher prevalence in Santa Cruz, RN (60.1%; 17.1%)^(^ 8 ^)^ and the SABE study conducted in São Paulo, SP (48.8%; 40.6%)^(^ 6 ^)^. Likewise, studies conducted in the United States (46.6%; 6.9%)^(^ 1 ^)^ and in Mexico (47%; 15.7%)^(^ 7 ^)^ report different results.

The association of pre-frailty and frailty with being 70 to 79 years old or 80 years old or older corroborates the findings of both Brazilian^(^ 5 ^,^ 8 ^)^ and international studies^(^ 1 ^,^ 6 ^-^ 7 ^)^. The influence of aging as a predisposition for the development of the frailty syndrome may be related to changes and decline taking place in multiple systems, due to the interaction of physiological mechanisms and pathological conditions^(^ 1 ^)^ with current and accumulated risks and functionality^(^ 4 ^)^. Nonetheless, even though aging predisposes to the frailty syndrome, not all elderly individuals are frail^(^ 16 ^)^ or pre-frail, suggesting there are common but not identical elements. Hence, it is believed that this syndrome presents more accentuated characteristics than those regarding the normative process of physiological aging^(^ 17 ^)^.

The pre-frailty condition was associated with the absence of a partner; a similar result was found among Mexican pre-frail and frail elderly individuals^(^ 7 ^)^. A longitudinal study conducted in São Paulo, SP reports female elderly individuals separated or divorced present mortality rates 82% and 35%, respectively, higher than that observed among their married counterparts^(^ 18 ^)^. In this sense, considering that the marital status is a component of the social support network of elderly individuals, we assume that, considering its complex interaction with clinical and social factors, the frailty syndrome sets in when there is rupture and/or absence of social ties^(^ 3 ^,^ 19 ^)^, considering a decline in physiological reserves^(^ 1 ^)^ and the existence of stressful events or factors.

As opposed to other Brazilian and international studies^(^ 1 ^,^ 5 ^-^ 6 ^)^, the variables: sex, housing, education and income were not associated with pre-frailty and frailty conditions. Other variables, such as being a woman, having low income and education, and living alone pose risks to the development of the frailty syndrome^(^ 1 ^,^ 6 ^-^ 7 ^)^, suggesting that disadvantages such as economic, educational and health deficits accumulated over the course of life may contribute to the problem^(^ 4 ^)^.

In this study, the condition of frailty was associated with hospitalization in the last year, a result that disagrees with that reported by a study conducted in Belo Horizonte, MG, which found this association among frail and pre-frail elderly individuals^(^ 5 ^)^. Frail individuals experience decreased ability to respond to stressful situations, a vulnerability that predisposes them to chronic diseases, anorexia, sarcopenia, osteopenia, cognitive deficit and disabilities, which justify their greater susceptibility to adverse outcomes, such as hospitalization^(^ 1 ^-^ 2 ^)^.

Additionally, intervening hospitalizations are strongly associated with mortality in the transition between states of frailty. Hospital environments may compromise the functional conditions of elderly individuals, hindering recovery from frailty and pre-frailty conditions. These findings show the need for actions to reduce hospitalizations due to evitable causes, preventive measures to avoid hospital complications^(^ 20 ^)^, and the implementation of care protocols that take into account admission, procedures, surgeries, time of hospitalization, hospital discharge and post discharge.

The pre-frailty and frailty conditions remain associated with a greater use of medication, especially among those taking 5 or more medications. This finding was also reported by an international investigation in which polypharmacy was associated with increased prevalence and incidence of frailty among community-dwelling elderly individuals^(^ 21 ^)^. Polypharmacy is considered a risk factor for frailty among the elderly^(^ 21 ^)^, in which the overlap of multiple medications, indiscriminate use of medications, and associated side effects^(^ 22 ^)^, may aggravate this condition. Moreover, the association between frailty and chronic diseases in this study indicates that the greater use of medications may reflect the manifestation of comorbidities. 

This study's results include the association between pre-frailty and frailty and morbidities, corroborating Brazilian^(^ 5 ^,^ 8 ^)^ and international^(1-2,7) ^studies. Frailty and chronic diseases function as modulators of an individual's health, suggesting that understanding the presence or absence of these conditions may favor the representation of physiological reserves in old age^(^ 22 ^)^.

Frailty may enhance the development or progression of chronic diseases, possibly because of decreased levels of activity or the progression of other conditions that affect mechanisms responsible for homeostasis, such as inflammatory processes and sympathetic-parasympathetic balance^(^ 2 ^)^. Additionally, frail elderly individuals experience increased vulnerability to stressful events, such as the manifestation of pathological processes due to low energetic reserves and/or their inefficient use, which result from a pathological response of the limited physiological reserve of this syndrome^(^ 22 ^)^.

Disability or dependence for the performance of instrumental activities of daily life was associated with the conditions of frailty and pre-frailty, however, disability to perform basic activities of daily life was only associated with frailty. Brazilian^(^ 5 ^,^ 8 ^)^ and international^(^ 1 ^,^ 7 ^,^ 23 ^)^ studies also report associations between frailty and disability to perform BADL and IADL. Despite distinctions in their theoretical conception^(^ 2 ^)^ and confusion regarding the definition of frailty and disability due to the similarity between their adverse outcomes, frailty predicts disability among elderly individuals^(^ 23 ^)^, which in turn, is seen as a outcome or contributing factor, potentially aggravating frailty and comorbidities^(^ 2 ^)^. Therefore, there is a need for care actions directed to the organization of healthcare services, family and society to cope with this scenario, in order to delay or alleviate functional decline in elderly individuals, taking into account frailty conditions, and specially pre-frailty, to promote an active aging process^(^ 5 ^)^.

Frail elderly individuals are 80% more likely to develop depressive symptoms. This association was also verified in other studies addressing pre-frail (OR=3.82; CI=3.72-3,93) and frail (OR=11.23; CI=10.89-11.58) Mexican individuals^(^ 7 ^)^, as well as pre-frail (OR=1.77; CI=1.16-2.71) and frail (OR=2.62; CI=1.23-7.02) Brazilians^(^ 5 ^)^.

The complex and two-way causal nature between frailty and depression remains unknown. The presence of depressive symptoms may constitute a risk factor for this syndrome since changes in behavior, activity level, or social commitment may contribute to the decline of one's functional state and frailty. On the other hand, depressive symptoms may represent the early manifestation of frailty, worsening one's mood and depression due to the syndrome. Additionally, these conditions may overlap considerably, possibly indicating that somatic complaints are symptoms of diseases associated with the frailty syndrome^(^ 24 ^)^.

Pre-frail and frail elderly individuals present 67% and 82% more chances, respectively, of presenting a negative health perception, a result that agrees with that reported by other studies^(^ 6 ^,^ 8 ^)^. Given characteristics inherent to the condition of frailty and pre-frailty, such as decreased ability to respond to stressful conditions and greater susceptibility to adverse events (e.g. the progression of diseases), it is believed that these aspects justify such an association. On the other hand, it is possible that the perception of elderly individuals with regard to adverse events experienced over the course of life^(^ 8 ^)^, such as: personal experiences, goals, and mechanisms used to overcome disappointments and failures, may predispose to the frailty syndrome^(^ 8 ^)^.

## Conclusion

The conditions of frailty presented a percentage higher than that reported by Brazilian studies and associated with health-related variables. The study revealed 313 (32.7%) non-frail elderly individuals, 522 (55.4%) pre-frail individuals and another 128 (12.8%) frail ones. The factors associated with pre-frailty and frailty conditions, respectively, were: being 70├79 years old and 80 years old or older; the use of 1├4 medications and 5 or more; higher number of morbidities, functional disability for instruments activities of daily living and negative perception of life. The absence of a partner was associated with pre-frailty, while hospitalization in the last year, functional disability to perform basic activities of daily life and indication of depression were associated with frailty.

The study's limitations included its cross-sectional design, which does not permit establishing a relationship of causality with the self-reported morbidities. The results of this investigation, however, contribute to deepen knowledge of the frailty syndrome among Brazilian elderly individuals and support the planning and implementation of interventions and care actions directed to this condition to prevent, revert or impede its progression.
